# Digital Technologies for Schizophrenia Management: A Descriptive Review

**DOI:** 10.1007/s11948-021-00302-z

**Published:** 2021-04-09

**Authors:** Olga Chivilgina, Bernice S. Elger, Fabrice Jotterand

**Affiliations:** 1 Institute of Biomedical Ethics, University of Basel, Basel, Switzerland; 2 Unit of Health Law & Humanitarian Medicine At the Institute for Legal Medicine, University of Geneva, Geneva, Switzerland; 3 Center for Bioethics and Medical Humanities, Institute for Health and Equity, Medical College of Wisconsin, Milwaukee, USA

## Abstract

**Abstract:**

While the implementation of digital technology in psychiatry appears promising, there is an urgent need to address the implications of the absence of ethical design in the early development of such technologies. Some authors have noted the gap between technology development and ethical analysis and have called for an upstream examination of the ethical issues raised by digital technologies. In this paper, we address this suggestion, particularly in relation to digital healthcare technologies for patients with schizophrenia spectrum disorders. The introduction of digital technologies in psychiatry offers a broad spectrum of diagnostic and treatment options tailored to the health needs and goals of patients’ care. These technologies include wearable devices, smartphone applications for high-immersive virtual realities, smart homes, telepsychiatry and messaging systems for patients in rural areas. The availability of these technologies could increase access to mental health services and improve the diagnostics of mental disorders.

**Additional Instruction Abstract:**

In this descriptive review, we systematize ethical concerns about digital technologies for mental health with a particular focus on individuals suffering from schizophrenia. There are many unsolved dilemmas and conflicts of interest in the implementation of these technologies, such as (1) the lack of evidence on efficacy and impact on self-perception; (2) the lack of clear standards for the safety of their daily implementation; (3) unclear roles of technology and a shift in the responsibilities of all parties; (4) no guarantee of data confidentiality; and (5) the lack of a user-centered design that meets the particular needs of patients with schizophrenia. mHealth can improve care in psychiatry and make mental healthcare services more efficient and personalized while destigmatizing mental health disorders. To ensure that these technologies will benefit people with mental health disorders, we need to heighten sensitivity to ethical issues among mental healthcare specialists, health policy makers, software developers, patients themselves and their proxies. Additionally, we need to develop frameworks for furthering sustainable development in the digital technologies industry and for the responsible usage of such technologies for patients with schizophrenia in the clinical setting. We suggest that digital technology in psychiatry, particularly for schizophrenia and other serious mental health disorders, should be integrated into treatment with professional supervision rather than as a self-treatment tool.

## Introduction–a Boom of New Technologies

Mhealth software is a rapidly growing market, with a significant part being represented by technologies for mental health, including technologies for schizophrenia spectrum disorders. (Chivilgina et al., 2020). Schizophrenia is one of the most burdensome psychiatric disorders, affecting up to 1% of the population worldwide (Saha et al., 2005). It is a heterogeneous disorder, which means that schizophrenic patients may experience not only psychotic symptoms but also mood instabilities, disturbances of intentionality, or cognitive deficits.

In light of the quick technology development, there is a panoply of ethical questions related to the adoption of such technology among patients with schizophrenia, such as (1) the lack of evidence on efficacy and impact on self-perception, which means that not every technology can provide beneficence to patients; (2) the lack of clear standards for the safety of their daily implementation,which can potentially lead to harm; (3) unclear roles of technology and a shift in the responsibilities of all parties; (4) no guarantee for confidentiality and privacy of sensitive data; (5) the lack of a user-centered design that meets the particular needs of patients with schizophrenia.

We begin our analysis with an examination of Big Data tools in mental healthcare and their ethical implications for psychiatry broadly and with regard to the implementation of digital technologies for patients with schizophrenia. First, we address the impact of digital technologies on patient identity and self-perception. This is an important issue for patients with schizophrenia since these individuals are delusional and often experience identity disturbance and blurred self-concept or lose touch with reality (Kallai et al., 2018). Second, we examine the implications of digital technologies for clinical practice, particularly with regard to how they might reconfigure the clinician-patient relationship while recognizing the effects of psychiatric disorders on autonomy and decision-making capacity. The technology-clinician-patient triangulation, although not a new issue per se, requires clarification of the boundaries of responsibility between the patient and the clinician when potentially disruptive technologies are introduced into the clinical context.

## Methods

### Data Search and Extraction

A systematic literature review was performed to retrieve a comprehensive and the up-to-date list of digital technologies with application to schizophrenia. Published studies were identified for the period between January 2003 and October 2019 in several databases: Scopus, PubMed, Web of Science, PsycINFO, PSYNDEX, ACM Digital Library, PsycArticles and Embase. We developed the following search strategy using Boolean logic: (‘Mobile apps’ OR ‘digital healthcare’ OR ‘mHealth’) AND ‘schizophrenia’ AND (‘management’ OR ‘treatment’ OR ‘efficacy’ OR ‘recommendations’).

Thematic inclusion criterion was an original research study focusing only on digital health technologies that claimed to be designed for people with schizophrenia spectrum and other psychotic disorders. In our review, we excluded telemedicine and SMS-messaging, as they do not represent an innovative technology. This strategy resulted in 1088 abstracts. Subsequently, following the Preferred Reporting Items for Systematic Reviews and Meta-Analyses (PRISMA) framework, four steps of filtering were performed (Moher et al., 2015): additional records identification through secondary sources, duplicates removal (both software-assisted and manual), eligibility assessment and inclusion. To minimize subjective biases, each stage of review was performed by at least two authors independently from each other.

### Data Analysis and Synthesis

An in-depth review of full-text articles included in the synthesis (n = 264) was performed. Our quantitative document analysis consisted of three sequential steps. To achieve the purpose of the review, we examined the presence of ethically-relevant considerations for each digital technology. During this phase, ethically relevant keywords and statements were searched in the full texts of all reviewed articles, using both software-guided keyword search (software used: EndNote X9) and unguided full-text review. Then, using qualitative thematic analysis we clustered all retrieved ethical considerations into main thematic families. (Vaismoradi et al., , 2013, 2016). Based on thematic affinity, our analysis identified four main thematic families: (1) privacy and confidentiality, (2) user-centered design, (3) patient identity, self-perception, and (4) patient-physician relationship. Each thematic family was further classified into sub-families relative to specific sub-components of the main ethical theme. When the same technology description contained more than one ethical consideration, all considerations were allocated to their respective thematic families.

The language used to describe the four thematic families was partly grounded on the principles of biomedical ethics(Beauchamp & Childress, 2009) and adapted to the specific context of digital technologies for schizophrenia.

We have chosen to classify autonomy, beneficence and responsibility as sub-component of patient-physician relationship rather then an independent categories, in accordance with the growing literature on patient-physician relationhip (Balint & Shelton, 1996; Kilbride & Joffe, 2018).

### From Big Data to Personalized Psychiatry

In the current era of rapid technological development, personal medical data are available from different sources. In the context of psychiatry, sensitive medical information about mental conditions is collected by hospitals and a number of digital devices. Hospital data, including genetic data, are subsequently stored in electronic healthcare records (EHRs); they can be combined with data from digital devices and used for analysis under the umbrella term of Big Data (Thinking big in mental health, 2018). Big Data is characterized by three features (the 3 Vs: big volume, high velocity and variety (Torous et al., 2015)) and poses paramount ethical issues regarding data sharing and data privacy. Digital devices are able to gather a vast **volume** of data, which may consist of so-called “active” data, which is generated by a patient’s active involvement such as taking surveys or questionnaires, and “passive” data, which refers to data that are generated without the patient’s involvement, such as GPS and accelerometer data or communication logs from voice calls and text messages. **Variety** refers to the diversity of gathered behavioral data and the innovative digital tools that can obtain and analyze different types of data wirelessly, such as physical parameters, neuroimaging or behavioral data. Real-time data collection creates new possibilities for monitoring health conditions. High-**velocity** data allow physicians to receive 24-h-a-day information about patients’ conditions and to identify signs of psychosis exacerbation and predict disease dynamics.

These opportunities provide new and useful knowledge that may improve treatment in psychiatry. For instance, genome-wide association studies in conjunction with Big Data analysis promise potential benefits for research and clinical practice, such as predicting illness or identifying overlap with other psychiatric disorders (Corvin & Sullivan, 2016; Diana O. Perkins et al.). Big Data technology has also been employed with the aim of enhancing the development of personalized psychiatry*.* Big Data can help in predicting individual treatment responses or risks, understanding how a condition manifests in particular individuals and designing interventions tailored to patients’ specific needs. The potential benefits of these technologies should not obscure the ethical challenges they raise in clinical practice, such as privacy and confidentiality, which we address in the next section.

### Ethical Issues–a Panoply of Challenges

Among technologies for mental health, there is a broad spectrum of digital technologies for schizophrenia, including mobile applications, computer programs, online therapies, virtual realities, and smart homes that target various symptoms and mechanisms of the disease. Our recent literature review of current mobile health technologies under development or available on the market revealed a lack of consideration of the ethical implications associated with their use in patients with schizophrenia spectrum diseases (Chivilgina et al., 2019). In what follows, we provide an in-depth analysis of the main ethical issues arising from the use of mobile health technologies with a particular focus on patient identity, self-perception, autonomy, decision-making, and the patient-physician relationship, privacy and confidentiality and user-centered design.

## Privacy and Confidentiality

To analyze the issue of privacy and confidentiality in Big Data, we refer to the concept of the “extended digital phenotype” (Loi, 2019). This interpretation of Dawkins’s idea considers personal medical information part of an individual; therefore, data protection and ownership frameworks should have the same moral consideration as, for instance, biological tissues. Data ownership by different parties and data flow to insurance companies may compromise patients’ control over their own data. According to the General Data Protection Regulation (GDPR) ('The EU General Data Protection Regulation (GDPR),' May 25th, 2018) in Europe and the Health Insurance Portability and Accountability Act (HIPAA) ('Health Insurance Portability and Accountability Act of 1996,' 08/21/1996) in the US, each person owns his or her information. These regulatory bodies establish a right to restrict the flow of data and a right “to be forgotten”, that is, the right to have data erased, which is not, we should stress, always possible.

Despite the HIPAA and GDPR regulations and according to a systematic review we completed recently (Chivilgina et al., 2019), 35% of mobile technologies do not have data protection provisions. Except for the properties of programs themselves for data exchange, users are not always aware of the risks associated with these technologies and therefore contribute to the increase of cybersecurity risks, such as the transmission of personal information without encryption (Huckvale et al., 2015) or malware installations (Boulos et al., 2014). Another issue is the use of social networking apps, which can help to enhance social behavior (Alvarez-Jimenez et al., 2019; Webber & Fendt-Newlin, 2017). Improving social capacity is an important aspect of behavioral therapy for people with schizophrenia. Social networking apps, however, increase the risks of addiction to device usage, stigmatization, and cyberbullying (Urano et al., 2020). Following a psychotic relapse, a patient may neglect the impact of social media use and post messages that may have long-term reputation, privacy, and legal implications. Consequently, mechanisms are needed to ensure the privacy and confidentiality of personal information, as are strategies to educate patients about these important issues. It is important to note that privacy and confidentiality also mean the right to freedom of thought: individuals have the right to control their own mental processes, cognition, and consciousness and keep them private. Expectations about confidentiality are often higher in psychiatry than in other disciplines because of the nature of the information gathered. A psychiatric condition is sometimes more than a diagnosis; it has a particular meaning, carries particular emotions and is interwoven with the narrative of a person. The data collected contain information about the personality and intimate details such as private thoughts or fantasies. For this reason, confidentiality represents a main challenge in the implementation of technologies such as BCIs.

### User-Centered Design

An important consideration in the development of digital technologies is the so-called “user-friendly design”. In this approach to technology development, special arrangements are deemed necessary in the design, such as adapted page complexity, navigational simplicity, and comprehensibility, to accommodate the needs of people with serious mental health conditions (Rotondi et al., 2017). An interactive, attention-grabbing form of mobile applications causes high engagement, so digital technology may be used to provide comprehensive information, increase awareness of mental health diseases and serve as a first step for those who have avoided mental health care in the past. The same application could also be a useful tool to help improve low adherence to antipsychotic medication among patients with schizophrenia. Adherence remains one of the main problems in treatment and leads to decompensation or exacerbation of symptoms, relapse and rehospitalization (Ascher-Svanum et al., 2006). Moreover, technologies that stimulate responses and feedback among patients improve the decision-making process and accelerate treatment results. According to our previous analysis (Chivilgina et al., 2019), only 56% of technologies have a user-centered approach that includes (1) a user-centered design encompassing consideration about possible cognitive deficit, lack of engagement, experience of stigma, responsiveness to treatment, and provided tutorials and (2) online support for technology. In light of these issues, it appears that the design of digital technologies is not always adapted to the needs of patients suffering from mental illnesses. The term “user-centeredness” includes more than just usable interfaces. It is also about meeting the unique needs of specific patients and having their best interests at heart. Every patient has a unique disease manifestation that requires a personalized approach to improve the patient’s mental condition.

## Digital Technologies and Their Impact on Patient Identity and Self-Perception

The impact of digital technologies on patient identity and self-perception is still unclear, but this challenge should not deter us from critical analysis. According to the International Statistical Classification of Diseases and Related Health Problems 10th Revision (ICD–10), *distortions of thinking and perception and affects that are inappropriate or blunted* are central to the conceptualization, definition, and identification of schizophrenia (International Statistical Classification of Diseases and Related Health Problems 10th Revision (ICD-10)-WHO Version for: 2016, 2016). These include a heterogeneous group of abnormal patterns, such as bizarre delusions (consisting of delusions of thought insertion, thought withdrawal, being controlled, thought broadcasting, and delusions of mind reading), disorganization (grandiose delusions among other non-delusion symptoms), and non-bizarre delusions (consisting of delusions of persecution, reference, jealousy, and sin/guilt) (Kimhy et al., 2005). The cognitive and neurobiological mechanisms underlying delusional ideation are still under investigation. Anomalous self-experiences often occur as part of a confabulation. Therefore, patients with schizophrenia may present an unstable identity or changes in identity compared to healthy subjects (Boulanger et al., 2013). Identity is a very broad term that includes social, national, ethnic and other types of identity. In this paper, we draw upon Erikson’s definition of personal identity, or self-perception, in which identity reflects a subjective sense as well as an observable quality of personal sameness and continuity and the boundaries between “me” and the world (Erikson, 1970).

Some digital technologies, called virtual realities, are capable of giving their users the impression that they are inside a simulated space. These technologies could be widely used for research purposes to investigate the mechanisms of abnormal rationality because they allow us to assess patients’ behavior in particular environments, to provoke symptoms if their trigger is known and to measure symptom severity (Bekele et al., 2017; Canty et al., 2017; Han et al., 2014; Hesse et al., 2017; Mohammadi et al., 2018; Salgado-Pineda et al., 2016; van Bennekom et al., 2017). The clinical implementation of virtual/augmented reality (VR/AR) technologies in psychiatry and psychotherapy aims to teach patients coping skills that can be transferred from a virtual environment to their daily life. The improved technological capabilities introduce highly immersive VRs that can be implemented for severe debilitating paranoia and anxiety (Broome et al., 2013; Freeman et al., 2016), brief social skills interventions (Bekele et al., 2014; Rus-Calafell et al., 2014), cognitive training (Chan et al., 2010), and rehabilitation (Sohn et al., 2016).

The same therapeutic mechanism is used in animated conversational agents (relational agents or avatars), which are computer-animated humanoid characters that can simulate face-to-face conversation and can be used in psychotherapy. A computerized agent might be beneficial in enhancing medication adherence, for instance (Bickmore et al., 2010). Alternatively, an appropriate avatar of the persecutory voice can be constructed by the user and utilized as a therapeutic instrument by psychiatrists for the treatment of auditory hallucinations (Leff et al., 2013). Surprisingly, this approach of facilitating a dialogue between the patient and an avatar that represents the patient’s persecutory voice shows equivocal results in several studies (Dellazizzo et al., 2018; Fernandez-Caballero et al., 2017; Leff et al., 2013): although participants’ drop-off rate due to side effects is high, the method is extremely effective for those who can tolerate it and gain control over the persecutory ideation by talking to a therapist who is telepresent behind the avatar. However, reports on the impact of such technologies on patients’ identity and self-perception remain scarce. This type of technology poses more questions than answers. What is human embodiment in the virtual world? Is it a digital identity? Is virtual reality a space for individuals to express themselves without the consequences that would result in the real world, or should there be equivalent responsibility for and sanctioning of violence against other digital agents? How should one deal with inequalities and discrimination that occur online? These questions aim to stimulate further reflections but, due to the limited scope of the paper, cannot be addressed here. It should be noted that these questions are also difficult to address due to a lack of evidence and outcome data because of the restricted implementation of VR in mental health and limited research on its effects, risks and benefits.

The answers to practical questions such as whether VR technologies can cause or treat delusions or improve or deepen dysfunctional ways of thinking in some individuals as well as the duration and severity of these effects remain unclear because there are no studies on the long-term outcomes of VR therapy. Theoretically, virtual realities may have an unpredictable impact on people who may have an incorrect perception of themselves and “normal” reality. Due to the high immersiveness of some virtual realities and frailty of body representation, virtual experiences may have possible impacts on identity and self-perception in patients with mental health diseases, including, as proposed by Kellmeyer, agential uncertainty, a feeling of loss of control and unease about one’s (sense of) agency, phenomenological unease, self-alienation or epistemic uncertainty (Kellmeyer, 2018). Therefore, to establish safe environments, further examination is needed.

Another issue that impacts self-perception is how the gathering of “passive” data from sensors may exacerbate paranoid thoughts. For instance, several studies reported that participants used their phones only on “airplane mode” to avoid being tracked, deleted the program, lost, pawned or broke their phones or requested a replacement device (Batink et al., 2016).

## Impact of Technology on Clinician-Patient Interactions

Clinician-patient relationships have particular importance in psychiatry, especially in psychotherapy. Emerging technologies enhance, refine, and challenge clinician-patient relationships in many ways. The most important aspect is the potentially disruptive nature of these technologies in challenging and potentially transforming the clinician-patient encounter through a triangulated clinician-patient-technology collaboration or through a process in which technology may even substitute and replace a therapist, leaving the patient mostly alone in the digital world. To maintain a beneficial therapeutic relationship, it is crucial to understand the impact of technologies on patient autonomy, informed consent, beneficence, and fidelity and to draw new frames for responsibility.

## Autonomy and Informed Consent

Many authors recognize autonomy as a core philosophical concept in psychiatry (Paul Hoff, 2017). Indeed, psychiatry is a medical specialty in which patient autonomy has been neglected for a long time and coercion has been warranted by different ideologies. For instance, psychiatry was used for political means in the Soviet Union (van Voren, 2010) and by eugenic physicians during the Nazi era who conducted unethical experiments and sterilized and murdered individuals with schizophrenia (Strous, 2007). Even in our time, people’s autonomy is being disrespected. As the recent scandal with Cambridge Analytica shows, psychological Data can be re-sold without any concent from its owners and misused for political campaigns by manipulating opinions and imprisoning freedom of thought. (Carole Cadwalladr and Emma Graham-Harrison, Sat 17 Mar 2018). Data transfer without explicit consent is happening on a large scale: as it was revealed by Privacy International, many popular websites about depression in France, Germany and the UK shared user data with advertisers, data brokers and large tech companies, while some depression test websites provided the users’ answers and test results to third parties (The Privacy International, 2019).

Despite the recognition that individuals should have the freedom to make their own choices about their lives, there are different degrees of patient autonomy in clinical practice. Sometimes the ethical presumptions are non-transparent due to numerous practical challenges in clinical routine or when the decision-making capacity of a patient is limited. Patients are vulnerable due to their health status and lack of medical knowledge, which makes them dependent on the expertise of clinicians (Jotterand et al., 2016). Patients with mental health diseases are even more vulnerable because of the stigma associated with mental disorders and possible disabilities that affect decision-making capacity (Wang et al., 2017). In patients with schizophrenia, decision-making capacity may be compromised during acute psychotic episodes temporarily or even permanently. The latter case represents a basis for imposing restrictions on legal capacity. Thus, psychiatry remains a field where autonomy constraints can sometimes be justified by respect-based and beneficence-based arguments. Paternalistic approaches, such as involuntary hospitalization, might be implemented for patients who endanger themselves or other people. When treating unconscious patients, the wishes of patients should be respected based on what they communicated prior to their current state. When patients lack capacity due to a lack of cognitive abilities, patients’ proxies are sometimes presumed to make a decision in the patient’s best interest (relational autonomy), particularly if these family connections have a special meaning for the patient or if the decisions may affect the well-being of other family members. Finally, the most challenging phenomenon that psychiatrists often face is attenuated ambiguity aversion of schizophrenia patients (Pedersen et al., 2017) or possible changes in the individual’s core identity (Seeman, 2017), as discussed above. In that case, some skepticism exists regarding respect for personal autonomy while distinguishing and protecting “authentic” from “imposter” selves (Radoilska, Jul 2015).

The introduction of new technologies raises novel issues in the balancing act of supporting patient autonomy vs. paternalistic approaches to psychiatric patients with impaired decision-making. To start with the most encouraging aspects, these technologies empower patients to be autonomous decision-makers and to engage with multimedia tools. Patients who have access to appropriate technology can receive therapies that are poorly integrated into clinical treatment due to limited funding and inadequately trained staff.

In practice, however, technology can be a powerful tool that impacts autonomy in many ways. Deliberate self-monitoring programs, such as FOCUS (Ben-Zeev et al., 2013), refer to the individualistic model. This means that the patient initiates, continues and terminates the use of the technology by his/her own wish without clinical supervision. Surprisingly, an analysis by Singh et al. showed that most mobile applications did not react when a user entered potentially dangerous health information, e.g., selecting “yes” for “feeling suicidal” or entering extremely abnormal values for blood glucose levels (Many Mobile Health Apps Target High-Need, High-Cost Populations, But Gaps Remain, 2016). In the case of danger, the inability of the device to recognize threat to users shows that technology is inadequate to ensure non-maleficence. Non-maleficence, or the “do no harm” principle, lies at the heart of bioethics and medicine. It provides a moral basis to maintain trust between the patient and the physician. When patients decide to trust the monitoring of their medical condition or medical adherence to medical professionals, they rely on the safety of the treatment. If technologies developed for self-use exclude medical personnel from the therapeutic alliance, no harm should occur. Therefore, any technology intended for people with schizophrenia needs to be not only user-friendly but also a responsible interface that will ensure communication with healthcare professionals in case of any existing danger to a patient, such as suicidality, psychotic exacerbation, or complications.

In contrast to self-monitoring apps, some new technology innovations may have an overly broad controlling potential that limits patient autonomy. For example, Health Smart Home is capable of enhancing in-home medical treatment (Mano et al., 2016). The system gathers data from sensors such as home cameras, a wristband that detects patients’ falls and irregular movements or frontal cameras from mobile phones and other devices. The health data collected are managed by the Decision Maker algorithm, which can alert nurses and/or relatives whenever necessary. The devices can not only gather information that patients report about their condition themselves, so-called “active” data, but may also collect ambient “passive” data, such as detecting shifts in geolocation patterns from the global positioning system (GPS), declines in physical activity, increased nighttime app use, or discontinuation of all smartphone use. This information may provide important insights for individualized treatment or clinical research, but patients with schizophrenia, like any other individuals, have a right to privacy; therefore, they should be informed about the use of their “passive” data and be able to stop it at any moment. The potential of such technologies to undermine patient autonomy may be even stronger than involuntary hospitalization. When a patient is placed in the hospital, his liberty of movement is restricted. Some technological innovations that are designed for symptom tracking are reminiscent of Big Brother from 1984 in their unrestricted access to observation of patients’ behaviors. For such technologies, we recommend the use of a consent form that does not leave room for misinterpretation or the excess collection of data. Additionally, we argue that medical devices for mental health should collect only restricted types of behavioral data, and these limits should be discussed with the patient.

Another example of compulsory technology is digital pills that require the patient to wear a sensor that confirms drug ingestion (Kane et al., 2013; Peters-Strickland et al., 2016; Rohatagi et al., 2016). This technology aims to promote patients’ best interests to maintain therapy compliance. Therefore, this technology uses beneficence as a ranking principle and conflicts with patient autonomy. In general, there are two types of technologies: paternalistic ones that act for the sake of beneficence and those that respect autonomy and provide more freedom and flexibility to patients. With this distinction, mental health specialists can provide an individualized approach to each patient in terms of autonomy because there is no perfect “golden mean” between paternalism and autonomy for all cases in psychiatry. Mental health specialists can carry out a clinical assessment to evaluate whether the patient has sufficient mental capacity or potential for each grade on independence.,

The impact of technology on autonomy and decision-making is consequently changing the usual process of consenting, and a great deal of uncertainty still exists regarding whether the same standards of electronic consent are required for people with mental health disorders. Consent is valid only if the patient is competent, and during usual consent procedures, clinicians evaluate this competence. Most online apps do not have any instruments to access the mental state and cognitive capacity of users. In other words, the significance of consent in these digital online interventions becomes depreciated because they can access the content of a program even in the absence of legal capacity to consent. Moreover, electronic informed consent (e-consent) has been criticized because it does not achieve the same high standards as traditional informed consent. Patients may skip the page and press an agreement button without reading it (B et al., 2010). During an in-person encounter, the ability of a patient to make his or her own decision is clear to a therapist, but when patients use mobile software, it becomes challenging to evaluate their capability to understand the rules and to be truly informed about permission for personal data management.

Clinicians are usually obliged to educate the subject on consent and the content of treatment. A paper consent form may be long, but healthcare professionals clarify which procedures will be performed, what the purpose of the intervention is, how it affects the patient, and whether there are any other options as well as the fact that the patient can always interrupt the procedure and “opt out”. Electronic informed consent is usually written in acceptance rules (user agreements), often in small font with dense and formal language (Martinez-Martin & Kreitmair, 2018). The complexity of informed consent and lack of readability are potential barriers to comprehension, so developers are responsible for creating technology that is easy to use and that provides online or peer tutorials. For this reason, we recommend explicit digital consent in applications for schizophrenia. This could be achieved, first, by integrating a standard for electronic informed consent for patients with possible cognitive deficits, including tools such as slowing down the consent process with interactive screens, bullet-point summaries of the most important risks or warnings, or providing video/audio content to clarify risks and benefits (National Institute of Mental Health, 2017. [2017–11-13].). Second, there is a need to assess the patient’s capacity to make decisions and his/her level of comprehension of the goals of the intervention, its terms of use and possible negative effects prior to undergoing a particular digital therapy. “Opt in” and “opt out” possibilities for sharing different types of personal information should be provided. Information about the psychiatric diagnosis or medication, psychological condition or personal life, sharing with other parties, and traceability of consent must be preserved in electronic consent to maintain the individual’s data ownership.

As suggested by the preceding analysis, digital technologies should provide a flexible framework for patients’ autonomy that does not involve spying on and controlling them or leaving them helpless in situations of crisis. In other words, digital tools, such as potential personalized therapy solutions, should provide patients with space for self-determination while guaranteeing their safety.

## Beneficence, Fidelity and Conflicts of Interest

In this section, we will compare user-technology interaction to a patient-doctor relationship to discuss how technology changes the concepts of beneficence and fidelity and introduces new stakeholders. In medical practice, doctors act in the best interest of their patients based on their medical knowledge and in line with clinical recommendations and the moral obligation to “do no harm”. The category of digital interventions is not homogenously compliant with regulatory recommendations. Currently, a mobile app can be developed and uploaded easily by anyone, and at first sight, this ubiquity of apps makes mental health accessible. However, this exposes people with mental health problems to unproven technical interventions without reliable scientific evidence. Direct-to-consumer applications that are developed without research may obscure danger to patients, contain incorrect information, give dangerous advice on treatment or lifestyle choices, or prevent patients from receiving proper treatment. In our previous review, we identified the scarcity of high-quality research studies on applications for schizophrenia due to a low number of participants. In addition, studies on these applications demonstrate a lack of comparison with control groups and unknown efficacy of long-term follow-up. Also a “digital placebo” effect (a placebo-like effect from the use of technologies, such as mobile apps) was not always considered.

Among the identified technologies for patients with schizophrenia, there are several artificial intelligence platforms, such as platforms for improving medication adherence in patients with schizophrenia (Bain et al., 2017). This technology presents a new set of ethical questions: can an algorithm have a notion of what beneficence is, and can it recognize the meaning of beneficence for a particular patient and act accordingly? These questions address a contentious issue that is continuously discussed in other papers (Mallah, 2017).

Concerning fidelity, it is essential that doctors gain the trust of their patients in face-to-face therapeutic alliance during long-term treatment by being open and honest with them. This appeals to one of the aspects of fidelity defined by Beauchamp and Childress, professional loyalty, which prioritizes the patient’s interests in two respects: 1. the professional effaces self-interest in any situation that may conflict with the patient interests, and 2. the professional favors patients’ interests over others’ interests (Beauchamp & Childress, 2009). Sometimes, patient beneficence may conflict with commercial interests. Health-related Big Data contains great value; therefore, an excessively wide range of gathered personal information might be used for the benefit of the company or “nudging”, influencing customer choices towards particular products, rather than for the direct medical benefits of patients. Because of a growing power of software companies that own Big Data, consent letter and software regulations should be accessed independently by multiple experts from different background, such as psychiatric professional associations and consultant experts, by government and international health organizations' supervision. Another potential conflict of interest is a lack of explicitness regarding financial reimbursement for the costs of digital treatments. Despite the large variety of apps that are commercially available, the efficacy or effectiveness of these apps is largely unknown and disputable. As we observed in our systematic review, among mobile interventions for schizophrenia, there is a scarcity of high-quality research studies on mHealth applications (Chivilgina et al., 2019). According to the principle of professional loyalty, when divided loyalties due to a conflict of interests appear, the well-being of patients should be prioritized. Technology is morally insensitive; it cannot act as a human with compassion and responsiveness or be loyal to the personal feelings of the patient. Therefore, a potential corollary of delegated treatment is a caring crisis. If patients do not feel that on the other side of the screen or in addition to the technology there is someone who is concerned about them as a person, then this crisis may ensue.

## Responsibility

Because technology cannot yet be morally sensitive, it cannot be responsible for the treatment process. In this part of the article, we further our ethical analysis with an issue of responsibility and explore its different dimensions. Because digital technologies are often quickly pushed onto the market without scientific validation (Joseph Conn, November 28, 2015), it is extremely complicated to find a useful program among bold marketing promises. Authors such as Torous insist that applications need to engender trust (Torous & Roberts, 2017). We argue that engendering trust must address the questions of responsibility and transparency. To move from a technology-driven to a user-centered approach, we need to define the role of technology in treatment and redraw the responsibilities of all stakeholders involved in the treatment of patients with schizophrenia.

Although there are laws that regulate medical software, they differ from country to counry, based on the various classifications of medical products,, so off-label use of medical products is possible. If technology is presented as a medical product to maximize benefits for the patient, it should be prescribed by the clinician according to the main symptoms of the disease. Currently, health technology assessments and the certification of applications for insurance fees are pressing topics. This indicates that technology coverage in psychiatry will grow. We argue that technologies for mental health should undergo not only a technical but also an ethical examination. Since direct-to-consumer technologies are available in Internet shops or can be delivered by mail, a gray zone exists for their use. There is also a growing do-it-yourself community in which individuals independently modify market-available technologies or build their own devices, such as transcranial brain stimulators (Kannon Yamada, November 14, 2014) despite some evidence this type of neuromodulation may impact task-related oscillatory activity in the frontal cortex (Singh et al., 2019). Ongoing effects from the at-home use of this type of technology are unknown; therefore, purposefulness of its use needs to be discussed with a doctor and the high-risk procedures should be performed by a healthcare specialist. For the good of the patient, implementation of digital technologies should be integrated within the medical treatment.

Medical software development should be a morally and legally accountable process that is performed with an abundance of caution when introducing it to the market and developing marketing materials and instructions for use.

Technological advances change the focus on responsibility and re-shapesthe therapeutic alliance.

The traditional shared decision-making model that is widespread in clinical settings assumes personal communication, offers choice, describes options, and discusses decisions (Elwyn et al., 2012). Within the decision-making process, this model shares responsibility between a doctor and a patient. There are technologies that maintain the same proportion of responsibility between doctors and patients as face-to-face therapy. However, the more that digital applications enhance autonomy, the greater the share of responsibility for decision-making that moves to patients. Patients become users, which has a different meaning: while a “patient” is a vulnerable person who needs a particular type of care (i.e., healthcare), a “user” is an active figure who is sufficiently responsible to use a particular product or service.

Doctors accurately, comprehensively, and objectively transmit information to patients according to the principle of veracity, which obliges them to be honest and tell the truth; this is not always the case in mobile applications. Patients who use technologies are free to create and fill out their own electronic health records; patients can read, report and access to the entire volume of their medical information anytime and anywhere. Consequently, each medical app user becomes responsible for reporting information about his medical condition and its validity.

Assistive technologies, such as smart homes (Mano et al., 2016) or robots (e.g., a pet-type robot for ball games and petting (Narita et al., 2016)), can reduce the burden faced by caregivers and healthcare specialists. The concepts of “self-monitoring” and “self-treatment” undermine roles in medical care. How patients can live independently with the help of digital solutions and the extent to which patients, caregivers, and healthcare systems can rely on the technology remains dependent on the severity of the disease. However, the accelerated use of technology does not fully replace the value of personal and medical care. Additionally, technology should not diminish clinicians’ responsibility for ensuring the best treatment for the patient (beneficence), respecting the patient’s self-determination (autonomy), and evaluating the patient’s decision-making capacity and consent. We argue for the need to maintain patient-relative relationships and clinician-patient relationships in the treatment of patients with mental health disorders. Just as good communication skills are required for doctors, digital medical technologies should be user-centered. In several articles, we observed that patients with schizophrenia are peer trained for apps when they are still in the hospital (Bucci et al., 2018; Forchuk et al., 2015; Verhagen et al., 2017). In contrast, one study recruited participants via the Internet without providing any assistance (Gulati et al., 2016). These approaches are hardly comparable since if people are contacted remotely, they may be less likely to report adverse effects. Nevertheless, for explicitness, we suggest personal contact in the beginning, which is a good solution to determine whether patients understand the app and its risks.

With the introduction of GDPR, patients become custodians of their own medical information. This raises an issue regarding the responsibility to educate vulnerable patients with fluctuating capacity about the risks and benefits of social media and digital technologies in general.

Overall, the concept of responsibility applied to technology for mental health is complex and multidimensional. Common dilemmas include responsibility, such as responsibility for the validity of the intervention, and maintaining standards of medical care, caring values, explicitness and transparency. There are unique aspects of responsibility related to mental health illness, as mentioned previously, including the responsibility for action in life-threatening situations, non-stigmatization of mental health conditions, and legal and current mental capacity to consent.

We believe that the involvement of all stakeholders, or the so-called participatory approach in technology development, is a key solution for increasing the responsiveness of technology to the needs of patients with mental health diseases.

## Discussion: Uncertainty and Call for Action

Disruptive technology provides many opportunities in mental health but also raises many ethical issues, and considerable uncertainty remains in its implementation. Due to these factors mistrust among health professionals and the public. First, there is a lack of transparent standards for mHealth applications. Internet advertising often offers apps based on financial benefit, and the visibility of applications in the App Store and Google Market is based on the star rating the app receives, not clinical efficacy. Adequate quality assessment should be provided and should be accessible to the public. There is a need for a decision-guiding framework from professional societies to help clinicians choose the right application for their patients.

Second, identifying the role and responsibilities of mobile apps in therapeutic relationships is disrupted by technology. All the issues that we discussed – data security gaps, safety, impact on self-perception and autonomy – may lead to mistrust in digital technology. A particularly confusing issue is the interfusion of roles and shifts in personal responsibilities. A person with mental health disease is vulnerable as a patient while simultaneously being authorized as a consumer who takes over many responsibilities from the moment he or she chooses the “I agree” button. Currently, regulations for wellness applications and data on any side effects of their amplification are scarce, thus it is problematic for health professionals to choose which clinical tasks they can delegate to mobile applications. Can they decrease hospital stays and encourage patients to use mobile apps in favor of reducing costs? Can they attempt to predict psychotic exacerbations by tracking and controlling their patients 24/7? Some authors propose that such apps should provide information regarding efficacy and safety claims, and the claims made in software advetrisements must be validated and the software companies should carry responsibility for misleading claims (Hsin & Torous, 2018). Another area of potential regulations need concerns legal responsibility in case of possible bad outcomes or side events (Armontrout et al., 2016).

Third, the high number of cases of data leakage and misuse of personal data on mental health has shown existing weaknesses of legal protection for confidentiality. There are concerns, that GDPR enforcement in Europe is still incomplete (Eddy, 2020). The recent analysis of privacy policies has revealed a lack of GDPR compliance in general health and medical apps (Mulder, 2019).

The studies by O’Loughlin et al. and Rosenfeld et al. have analyzed data policies of apps for dementia and depression (O'Loughlin et al., 2019; Rosenfeld et al., 2017). These papers have shown lack of comprehensive data policies in the apps, explaining the terms of data collection, data storage and data exchange. Moreover, many of the existing policies are vague and lack important information, such as details on encryption of data, password protection, and the ability to edit or delete entered information. The further research by Robillard has shown that the majority of mental health apps stated in the data policies that users’ information may be shared with third parties (Robillard et al., 2019).

Many recent cases have revealed that stealing or selling data on mental health and using it for digital phenotyping with commercial or political purposes is a real hazard. Thus, lack of clarity and transparency in the data governance and data sharing practices need to be addressed. Taken in account the fast pace of technological progress, proactive control, revision of data safety standards and independent audit of the software companies are needed for responsible implementation and management of mental health data. Additional efforts are needed to warranty data security in the process of data exchange with different parties. Some authors suggest that mental health apps have to document the processes they use to ensure the secure exchange of information between platforms (Torous et al., 2019).

Technology-mediated healthcare is becoming a growing reality in psychiatry, and it therefore maintains a relevant app ecosystem in mental health. Mobile mental health care applications are a potentially reliable mental health standard of care and a large step forward in the direction of personalized and high-precision medicine. To address the uncertainty in implementing mHealth in psychiatry, we need to heighten sensitivity to ethical issues. Additionally, we need to develop a responsible framework for furthering sustainable development in the digital technologies industry and the usage of such technologies for patients with schizophrenia in the clinical setting.

In light of these arguments, we would like to note that clinicians still play a significant role in supervising treatment. Digital technologies can upgrade psychiatric services and achieve better quality of care, but technology cannot be used as a substitute for a professional clinician's evaluation and advice until the problem of responsibility is solved. For these reasons, we argue that professional-patient relationships in psychiatry remain fiduciary. Very little is known about long-term outcomes of technology use in patients with severe mental health diseases (e.g., schizophrenia, schizoaffective disorder), so the usage of mobile apps among patients with these conditions remains the responsibility of psychiatrists based on the ethical principle of beneficence. Because patients might find apps and devices on their own through the Internet and private providers, the principle of beneficence implies that psychiatrists must actively ask their patients about the use of such technology. While many psychiatrists have been trained at a time when these technologies did not exist, beneficial care currently requires all psychiatrists to acquire minimal technology literacy related to their field in the interest of guiding their patients and, in the absence of proven benefits, preventing technology-related harm.

## References

[CR1] Alvarez-Jimenez M, Bendall S, Koval P, Rice S, Cagliarini D, Valentine L (2019). HORYZONS trial: protocol for a randomised controlled trial of a moderated online social therapy to maintain treatment effects from first-episode psychosis services. British Medical Journal Open.

[CR2] Chivilgina O, Wangmo T, Elger BS, Heinrich T, Jotterand F (2020). mHealth for schizophrenia spectrum disorders management: Asystematic review. International Journal of Social Psychiatry..

[CR3] Armontrout J, Torous J, Fisher M, Drogin E, Gutheil T (2016). Mobile mental health: navigating new rules and regulations for digital tools. Current Psychiatry Reports.

[CR4] Ascher-Svanum H, Faries DE, Zhu B, Ernst FR, Swartz MS, Swanson JW (2006). Medication adherence and long-term functional outcomes in the treatment of schizophrenia in usual care. The Journal of Clinical Psychiatry.

[CR5] Böhme, R, Stefan Köpsell (2010). Trained to accept?: a field experiment on consent dialogs. (*Paper presented at the Proceedings of the SIGCHI Conference on Human Factors in Computing Systems*, Atlanta, Georgia, USA)

[CR6] Bain EE, Shafner L, Walling DP, Othman AA, Chuang-Stein C, Hinkle J (2017). Use of a novel artificial intelligence platform on mobile devices to assess dosing compliance in a phase 2 clinical trial in subjects with schizophrenia. JMIR mHealth and uHealth.

[CR7] Balint J, Shelton W (1996). Regaining the initiative: forging a new model of the patient-physician relationship. JAMA.

[CR8] Batink T, Bakker J, Vaessen T, Kasanova Z, Collip D, van Os J (2016). Acceptance and commitment therapy in daily life training: A feasibility study of an mhealth intervention. JMIR mHealth and uHealth.

[CR9] Beauchamp TL, Childress JF (2009). Principles of Biomedical Ethics.

[CR10] Bekele E, Bian D, Peterman J, Park S, Sarkar N (2017). Design of a virtual reality system for affect analysis in facial expressions (VR-SAAFE); application to schizophrenia. IEEE Transactions on Neural Systems and Rehabilitation Engineering.

[CR11] Bekele, E., Bian, D., Zheng, Z., Peterman, J., Park, S., Sarkar, N. (2014). Responses during facial emotional expression recognition tasks using virtual reality and static IAPS pictures for adults with Schizophrenia. In: R. Shumaker, S. Lackey (Eds.), *Virtual, Augmented and Mixed Reality. Applications of Virtual and Augmented Reality. VAMR 2014. Lecture Notes in Computer Science* (Vol. 8526). Springer, Cham. 10.1007/978-3-319-07464-1_21.

[CR12] Ben-Zeev D, Kaiser SM, Brenner CJ, Begale M, Duffecy J, Mohr DC (2013). Development and usability testing of FOCUS: a smartphone system for self-management of schizophrenia. Psychiatric Rehabilitation Journal.

[CR13] Bickmore TW, Puskar K, Schlenk EA, Pfeifer LM, Sereika SM (2010). Maintaining reality: Relational agents for antipsychotic medication adherence. Interacting with Computers.

[CR14] Boulanger M, Dethier M, Gendre F, Blairy S (2013). Identity in schizophrenia: a study of trait self-knowledge. Psychiatry Research.

[CR15] Boulos MNK, Brewer AC, Karimkhani C, Buller DB, Dellavalle RP (2014). Mobile medical and health apps: state of the art, concerns, regulatory control and certification. Online Journal of Public Health Informatics.

[CR16] Broome MR, Zanyi E, Hamborg T, Selmanovic E, Czanner S, Birchwood M (2013). A high-fidelity virtual environment for the study of paranoia. Schizophr Research and Treatment.

[CR17] Bucci, S., Barrowclough, C., Ainsworth, J., Machin, M., Morris, R., Berry, K., et al. (2018). Actissist: Proof-of-concept trial of a theory-driven digital intervention for psychosis. *Schizophrenia Bulletin*, 44(5), 1070–1080. http://ovidsp.ovid.com/ovidweb.cgi?T=JS&CSC=Y&NEWS=N&PAGE=fulltext&D=psyc15&AN=2018-42793-020. 10.1093/schbul/sby032 PMC6135229 29566206

[CR18] http://sfx.metabib.ch/sfx_locater?sid=OVID:psycdb&id=pmid:&id=doi:10.1093%2Fschbul%2Fsby032&issn=0586-7614&isbn=&volume=44&issue=5&spage=1070&pages=1070-1080&date=2018&title=Schizophrenia+Bulletin&atitle=Actissist%3A+Proof-of-concept+trial+of+a+theory-driven+digital+intervention+for+psychosis.&aulast=Bucci&pid=%3Cauthor%3EBucci%2C+Sandra%3BBarrowclough%2C+Christine%3BAinsworth%2C+John%3BMachin%2C+Matthew%3BMorris%2C+Rohan%3BBerry%2C+Katherine%3BEmsley%2C+Richard%3BLewis%2C+Shon%3BEdge%2C+Dawn%3BBuchan%2C+Iain%3BHaddock%2C+Gillian%3C%2Fauthor%3E%3CAN%3E2018-42793-020%3C%2FAN%3E%3CDT%3EJournal+Article%3C%2FDT%3E.

[CR19] Canty AL, Neumann DL, Shum DHK (2017). Using virtual reality to assess theory of mind subprocesses and error types in early and chronic schizophrenia. Schizophr Research Cognition.

[CR20] Carole C, Graham-Harrison E (2018). Revealed: 50 million facebook profiles harvested for cambridge analytica in major data breach. The Guardian..

[CR21] Chan CL, Ngai EK, Leung PK, Wong S (2010). Effect of the adapted virtual reality cognitive training program among Chinese older adults with chronic schizophrenia: A pilot study. International Journal of Geriatric Psychiatry.

[CR22] Chivilgina O, Wangmo T, Elger BS, Heinrich T, Jotterand F (2020). mHealth for schizophrenia spectrum disorders management: A systematic review. International Journal of Social Psychiatry.

[CR23] Corvin A, Sullivan PF (2016). What next in schizophrenia genetics for the psychiatric genomics consortium?. Schizophrenia Bulletin.

[CR24] Dellazizzo L, Potvin S, Phraxayavong K, Lalonde P, Dumais A (2018). Avatar therapy for persistent auditory verbal hallucinations in an ultra-resistant schizophrenia patient: A case report. Front Psychiatry.

[CR25] Perkins DO, Loes Olde L, Barbee J, Ford J, Jeffries CD, Addington J (2019). Polygenic Risk Score Contribution to Psychosis Prediction in a Target Population of Persons at Clinical High Risk. American Journal of Psychiatry.

[CR26] Eddy, N. (2020 ). How EU Authorities See GDPR Effectiveness Two Years In. *eWeek*.

[CR27] Elwyn G, Frosch D, Thomson R, Joseph-Williams N, Lloyd A, Kinnersley P (2012). Shared decision making: A model for clinical practice. Journal of general internal medicine.

[CR28] Erikson, E. H. (1970). Autobiographic notes on the identity crisis. *Daedalus*, *99*(4), 730–759. 11609638

[CR30] Fernandez-Caballero A, Navarro E, Fernandez-Sotos P, Gonzalez P, Ricarte JJ, Latorre JM (2017). Human-avatar symbiosis for the treatment of auditory verbal hallucinations in schizophrenia through virtual/augmented reality and brain-computer interfaces. Frontiers in Neuroinformatics.

[CR31] Forchuk C, Reiss JP, O'Regan T, Ethridge P, Donelle L, Rudnick A (2015). Client perceptions of the mental health engagement network: A qualitative analysis of an electronic personal health record. BMC Psychiatry.

[CR32] Freeman D, Bradley J, Antley A, Bourke E, DeWeever N, Evans N (2016). Virtual reality in the treatment of persecutory delusions: Randomised controlled experimental study testing how to reduce delusional conviction. The British Journal of Psychiatry.

[CR33] Gulati G, Cornish R, Al-Taiar H, Miller C, Khosla V, Hinds C (2016). Web-based violence risk monitoring tool in psychoses: Pilot study in community forensic patients (Article). Journal of Forensic Psychology Practice.

[CR34] Han K, Shin J, Yoon SY, Jang DP, Kim JJ (2014). Deficient gaze pattern during virtual multiparty conversation in patients with schizophrenia. Computers in Biology and Medicine.

[CR35] 'Health Insurance Portability and Accountability Act of 1996' (08/21/1996).

[CR36] Hesse K, Schroeder PA, Scheeff J, Klingberg S, Plewnia C (2017). Experimental variation of social stress in virtual reality-Feasibility and first results in patients with psychotic disorders. Journal of Behavior Therapy and Experimental Psychiatry.

[CR37] Hsin H, Torous J (2018). Creating boundaries to empower digital health technology. BJPsych Open.

[CR38] Huckvale K, Prieto JT, Tilney M, Benghozi P-J, Car J (2015). Unaddressed privacy risks in accredited health and wellness apps: A cross-sectional systematic assessment. BMC Medicine.

[CR39] International Statistical Classification of Diseases and Related Health Problems 10th Revision (ICD-10)-WHO Version for: 2016. (2016). *Chapter V Mental and behavioural disorders (F00-F99)*.

[CR40] Joseph Conn (November 28, 2015 ). Easy on those apps: Mobile medical apps gain support, but many lack clinical evidence. *[Modern Healthcare]*. 26875253

[CR41] Jotterand F, Amodio A, Elger BS (2016). Patient education as empowerment and self-rebiasing. Medicine, Health Care and Philosophy.

[CR42] Kallai J, Rozsa S, Hupuczi E, Hargitai R, Birkas B, Hartung I (2018). Cognitive fusion and affective isolation: Blurred self-concept and empathy deficits in schizotypy. Psychiatry Research.

[CR43] Kane JM, Perlis RH, DiCarlo LA, Au-Yeung K, Duong J, Petrides G (2013). First experience with a wireless system incorporating physiologic assessments and direct confirmation of digital tablet ingestions in ambulatory patients with schizophrenia or bipolar disorder. Journal of Clinical Psychiatry.

[CR44] Kannon Yamada (November 14, 2014). Zap Yourself Smarter With This DIY tDCS Brain Stimulator. https://www.makeuseof.com/tag/build-tdcs-brain-stimulator/. Accessed.

[CR45] Kellmeyer P (2018). Neurophilosophical and ethical aspects of virtual reality therapy in neurology and psychiatry. Cambridge Quarterly of Healthcare Ethics.

[CR46] Kilbride MK, Joffe S (2018). The new age of patient autonomy: Implications for the patient-physician relationship. JAMA.

[CR47] Kimhy D, Goetz R, Yale S, Corcoran C, Malaspina D (2005). Delusions in individuals with schizophrenia: factor structure, clinical correlates, and putative neurobiology. Psychopathology.

[CR48] Leff J, Williams G, Huckvale MA, Arbuthnot M, Leff AP (2013). Computer-assisted therapy for medication-resistant auditory hallucinations: Proof-of-concept study. British Journal of Psychiatry.

[CR49] Loi M (2019). The digital phenotype: A philosophical and ethical exploration. Philosophy & Technology.

[CR50] Mallah, R. (2017). The Landscape of AI Safety and Beneficence Research. Input for Brainstorming at Beneficial AI 2017. *Beneficial AI 2017*.

[CR51] Mano LY, Faical BS, Nakamura LHV, Gomes PH, Libralon GL, Meneguete RI (2016). Exploiting IoT technologies for enhancing health smart homes through patient identification and emotion recognition. Computer Communications.

[CR52] High-Need and High-Cost Populations,But Gaps Remain(2016).Health Affairs, 35(12), 2310–2318.doi:10.1377, hlthaff.2016.0578. , 2016 Many Mobile Health Apps Target High-Need, High-Cost Populations, But Gaps Remain (2016). *Health Affairs*, 35(12), 2310-2318. doi:10.1377/hlthaff.2016.0578 10.1377/hlthaff.2016.0578 27920321

[CR53] Martinez-Martin N, Kreitmair K (2018). Ethical issues for direct-to-consumer digital psychotherapy apps: addressing accountability, data protection, and consent. JMIR mental health.

[CR54] Mohammadi A, Hesami E, Kargar M, Shams J (2018). Detecting allocentric and egocentric navigation deficits in patients with schizophrenia and bipolar disorder using virtual reality. Neuropsychological Rehabilitation.

[CR55] Moher D, Shamseer L, Clarke M, Ghersi D, Liberati A, Petticrew M (2015). Preferred reporting items for systematic review and meta-analysis protocols (PRISMA-P) 2015 statement. Systematic reviews.

[CR56] Mulder, T. (2019). Health Apps, their Privacy Policies and the GDPR. European Journal of Law and Technology, 2019, University of Groningen Faculty of Law Research Paper No.15/2020. Available at SSRN: https://ssrn.com/abstract=3506805.

[CR57] Narita S, Ohtani N, Waga C, Ohta M, Ishigooka J, Iwahashi K (2016). A pet-type robot Artificial Intelligence Robot-assisted therapy for a patient with schizophrenia. Asia-Pacific Psychiatry.

[CR58] National Institute of Mental Health ( 2017. [2017–11–13]. ). Opportunities and Challenges of Developing Information Technologies on Behavioral and Social Science Clinical Research.

[CR59] https://www.nimh.nih.gov/about/advisory-boards-and-groups/namhc/reports/opportunities-and-challenges-of-developing-information-technologies-on-behavioral-and-social-science-clinical-research.shtml Accessed.

[CR60] O'Loughlin K, Neary M, Adkins EC, Schueller SM (2019). Reviewing the data security and privacy policies of mobile apps for depression. Internet Interventions.

[CR61] Paul Hoff (2017). Ein Votum für den Dialog zwischen Psychiatrie und Philosophie. Autonomie, ein zentraler, aber sperriger Begriff der Psychiatrie (Review article). *Swiss Arch Neurol Psychiatr Psychother. 2017;168(06):175–182.*

[CR62] Pedersen A, Goder R, Tomczyk S, Ohrmann P (2017). Risky decision-making under risk in schizophrenia: A deliberate choice?. Journal of Behavior Therapy and Experimental Psychiatry.

[CR63] Peters-Strickland T, Pestreich L, Hatch A, Rohatagi S, Baker RA, Docherty JP (2016). Usability of a novel digital medicine system in adults with schizophrenia treated with sensor-embedded tablets of aripiprazole. Neuropsychiatric Disease and Treatment.

[CR64] Radoilska, L. (Jul 2015). Autonomy in Psychiatric Ethics. *The Oxford Handbook of Psychiatric Ethics*.

[CR65] Robillard JM, Feng TL, Sporn AB, Lai J-A, Lo C, Ta M (2019). Availability, readability, and content of privacy policies and terms of agreements of mental health apps. Internet Interventions.

[CR66] Rohatagi S, Profit D, Hatch A, Zhao C, Docherty JP, Peters-Strickland TS (2016). Optimization of a digital medicine system in psychiatry (Article). Journal of Clinical Psychiatry.

[CR67] Rosenfeld L, Torous J, Vahia IV (2017). Data Security and Privacy in Apps for Dementia: An Analysis of Existing Privacy Policies. The American Journal of Geriatric Psychiatry.

[CR68] Rotondi AJ, Spring MR, Hanusa BH, Eack SM, Haas GL (2017). Designing eHealth Applications to Reduce Cognitive Effort for Persons With Severe Mental Illness: Page Complexity, Navigation Simplicity, and Comprehensibility. JMIR Human Factors.

[CR69] Rus-Calafell M, Gutierrez-Maldonado J, Ribas-Sabate J (2014). A virtual reality-integrated program for improving social skills in patients with schizophrenia: a pilot study. Journal of Behavior Therapy and Experimental Psychiatry.

[CR70] Saha S, Chant D, Welham J, McGrath J (2005). A systematic review of the prevalence of schizophrenia. PLoS Medicine.

[CR71] Salgado-Pineda P, Landin-Romero R, Portillo F, Bosque C, Pomes A, Spanlang B (2016). Examining hippocampal function in schizophrenia using a virtual reality spatial navigation task. Schizophrenia Research.

[CR72] Seeman MV (2017). Identity and schizophrenia: Who do I want to be?. World journal of psychiatry.

[CR73] Singh A, Trapp NT, De Corte B, Cao S, Kingyon J, Boes AD (2019). Cerebellar Theta Frequency Transcranial Pulsed Stimulation Increases Frontal Theta Oscillations in Patients with Schizophrenia. The Cerebellum.

[CR74] Sohn BK, Hwang JY, Park SM, Choi JS, Lee JY, Lee JY (2016). Developing a Virtual Reality-Based Vocational Rehabilitation Training Program for Patients with Schizophrenia. Cyberpsychology, Behavior and Social Networking.

[CR75] Strous RD (2007). Psychiatry during the Nazi era: ethical lessons for the modern professional. Annals of general psychiatry.

[CR29] The EU General Data Protection Regulation, REGULATION (EU) 2016/679 OF THE EUROPEAN PARLIAMENT AND OF THE COUNCIL of 27 April 2016 on the protection of natural persons with regard to the processing of personal data and on the free movement of such data, and repealing Directive 95/46/EC (General Data Protection Regulation), Chapter 3.

[CR76] The Privacy International (2019). REPORT: Your mental health for sale. https://privacyinternational.org/node/3193.

[CR77] Thinking big in mental health (2018). Editorial. Nature Medicine.

[CR78] Torous J, Andersson G, Bertagnoli A, Christensen H, Cuijpers P, Firth J (2019). Towards a consensus around standards for smartphone apps and digital mental health. World psychiatry : official journal of the World Psychiatric Association (WPA).

[CR79] Torous J, Roberts LW (2017). Needed Innovation in Digital Health and Smartphone Applications for Mental Health: Transparency and Trust. JAMA Psychiatry.

[CR80] Torous J, Staples P, Onnela J-P (2015). Realizing the Potential of Mobile Mental Health: New Methods for New Data in Psychiatry. Current psychiatry reports.

[CR81] Urano Y, Takizawa R, Ohka M, Yamasaki H, Shimoyama H (2020). Cyber bullying victimization and adolescent mental health: The differential moderating effects of intrapersonal and interpersonal emotional competence. Journal of Adolescence.

[CR82] Vaismoradi, M., Jones, J., Turunen, H., & Snelgrove, S. (2016). Theme development in qualitative content analysis and thematic analysis.

[CR83] Vaismoradi M, Turunen H, Bondas T (2013). Content analysis and thematic analysis: Implications for conducting a qualitative descriptive study. Nursing & Health Sciences.

[CR84] van Bennekom MJ, de Koning PP, Denys D (2017). Virtual Reality Objectifies the Diagnosis of Psychiatric Disorders: A Literature Review. Front Psychiatry.

[CR85] van Voren R (2010). Political Abuse of Psychiatry—An Historical Overview. Schizophrenia Bulletin.

[CR86] Verhagen SJW, Berben JA, Leue C, Marsman A, Delespaul P, van Os J (2017). Demonstrating the reliability of transdiagnostic mHealth Routine Outcome Monitoring in mental health services using experience sampling technology. PLoS ONE.

[CR87] Wang S-B, Wang Y-Y, Ungvari GS, Ng CH, Wu R-R, Wang J (2017). The MacArthur competence assessment tools for assessing decision-making capacity in schizophrenia: A meta-analysis. Schizophrenia Research.

[CR88] Webber M, Fendt-Newlin M (2017). A review of social participation interventions for people with mental health problems. Social psychiatry and psychiatric epidemiology.

